# Real-world practices of hormone monitoring during ovarian stimulation in assisted reproductive technology: a global online survey

**DOI:** 10.3389/fendo.2023.1260783

**Published:** 2023-11-28

**Authors:** Noemie Sachs-Guedj, Roger Hart, Antonio Requena, Vanessa Vergara, Nikolaos P. Polyzos

**Affiliations:** ^1^ Reproductive Medicine, Dexeus University Hospital, Barcelona, Spain; ^2^ Department of Pediatrics, Obstetrics and Gynecology, Faculty of Medicine, Autonomous University of Barcelona, Barcelona, Spain; ^3^ Reproductive Medicine, University of Western Australia/Fertility Specialist of Western Australia, Perth, WA, Australia; ^4^ Reproductive Medicine, IVI Madrid, Madrid, Spain; ^5^ Faculty of Medicine and Health Sciences, Ghent University (UZ Gent), Gent, Belgium

**Keywords:** assisted reproductive technique, controlled ovarian stimulation, hormonal monitoring, ovarian hyperstimulation syndrome, ovulation trigger

## Abstract

**Objective:**

The aim of this study is to understand the global practice of routine hormonal monitoring (HM) during ovarian stimulation (OS) in the context of assisted reproductive technique (ART) treatment.

**Methods:**

An open-access questionnaire was available to 3,845 members of IVF-Worldwide.com from September 8 to October 13, 2021. The survey comprised 25 multiple-choice questions on when and how ultrasound (US) and hormone tests were conducted during ovarian stimulation OS. For most questions, respondents were required to select a single option. Some questions allowed the selection of multiple options.

**Results:**

In all, 528 (13.7%) members from 88 countries responded to the questionnaire. Most respondents (98.9%) reported using US to monitor OS cycles. HM was used by 79.5% of respondents during any of the cycle monitoring visits and was most commonly performed on the day of, or a day prior to final oocyte maturation. Overall, 87% of respondents claimed adjusting the dose of gonadotropin during OS, with 61.7% adjusting the dose based on hormonal levels. Oestradiol (E2) was the most frequently monitored hormone during all visits and was used by 74% of respondents for the prediction of ovarian hyperstimulation syndrome (OHSS). On or a day prior to ovulation triggering (OT), the number of respondents who measured progesterone increased from 34.3% in the second/third visit to 67.7%. Approximately one-third of respondents measured luteinizing hormone during all visits.

**Conclusion:**

Globally, most ART specialists (~80%) use HM, along with US, for monitoring OS, especially for the prevention of OHSS.

## Introduction

Ovarian stimulation (OS) in assisted reproductive technique (ART) cycles aims to procure an optimal number of mature oocytes, with a high probability of good quality embryos. The objective of optimal OS is to lead to an acceptable cumulative pregnancy rate (1). There are several phases of ART that can be customized for the best outcomes (2) including OS, ovulation triggering (OT), and luteal phase support (2, 3). Gonadotropin dose adjustment on days 4 to 6 of OS and at later time points during stimulation can be considered, based on a patient’s response (4). Dose reduction could also be important to prevent ovarian hyperstimulation syndrome (OHSS), which is the most critical and potentially life-threatening complication of ovulation stimulation (5).

Ovarian stimulation protocol involves adjustment of gonadotropin dose, addition of another gonadotropin (e.g., luteinizing hormone [LH]), modification of the type of gonadotropin, change of the planned agent for OT, or planning an elective ‘freeze-all embryos’ cycle (4). Before dose adjustment, a patient’s response to stimulation is evaluated by ultrasound (US) to monitor follicular development, and the evaluation may also include measuring serum hormone concentrations (2).

Typically, clinical practice guidelines recommend only US for monitoring to assess a patient’s response to OS. The 2019 guideline by the European Society of Human Reproduction and Embryology (ESHRE) stated that the addition of oestradiol (E2) measurements to US monitoring during OS is not recommended as it did not appear to decrease the probability of OHSS occurring, increase the probability of a clinical pregnancy, or the number of oocytes retrieved (6).

According to this guideline, the combination of E2, progesterone (P4), and LH monitoring was not recommended as it did not appear to increase the probability of pregnancy, the number of cumulus-oocyte complexes retrieved, or decrease the probability of OHSS or cycle cancellation. Further, the guideline also does not recommend adjustment of the gonadotropin dose in the mid-stimulation phase during OS. Nevertheless, it does mention that the decision on the timing of OT in relation to follicle size is multi-factorial. The factors proposed included the size of the growing follicle cohort, the hormonal data on the day of proposed trigger, the duration of ovarian stimulation, patient burden, financial costs, the experience of previous cycles, and organizational factors for the *in-vitro* fertilization (IVF) center (6). A systematic review and meta-analysis published in 2014 concluded that monitoring COS with the US alone is unlikely to significantly impact the probability of achieving a clinical pregnancy (7). However, the evidence presented was considered low-quality. The review also concluded that the number of oocytes retrieved with US monitoring alone is similar to the number of oocytes retrieved when monitoring with US and hormonal assessment; this evidence was considered moderate-quality. The review was inconclusive for the other outcomes and comparisons such as OHSS and miscarriage (7). Similarly, a Cochrane review in 2021 (8) concluded that evidence did not suggest that combined monitoring by US and serum oestradiol was more effective than monitoring by US alone regarding clinical pregnancy and OHSS. However, the evidence was considered low-quality for all comparisons.

It is believed that until now, no study has evaluated the attitude and practice of clinicians regarding HM during COS. Hence, this survey was conducted to understand the global practice of routine HM during OS in the context of ART treatment.

## Materials and methods

### Study design, size, duration

In a cross-sectional survey on the current practice of blood HM, we evaluated physicians’ attitudes towards blood HM during OS in the context of ART treatment. An open-access questionnaire was accessible to the members of IVF-Worldwide.com from September 8 to October 13, 2021, on IVF-Worldwide.com (9). An initial invitation and one reminder were sent to all 3845 registered members of IVF-Worldwide.com by email.

Data analysis was performed using Excel (Microsoft Inc., USA).

### Questionnaire

The survey comprised 25 multiple-choice questions. These questions enquired about tests performed during any of the cycle monitoring visits during OS for ART treatment (blood HM, US, timing, and frequency of prescribed tests) to prevent OHSS as well as to adjust the gonadotropin dose. For most questions, a single option was required to be selected by respondents. A small number of questions allowed the selection of multiple options.

## Results

A total of 528 out of 3845 (13.7%) members from 88 countries responded to this web-based survey (10); 39.3% of the respondents were based in Europe, 14% in Latin America, 9.1% in North America and 37.6% in Asia-Pacific and rest of the world. Most participants (87.9%) were clinicians and practiced reproductive medicine for more than 15 years (56.7%). Nearly half (46.2%) performed more than 500 oocyte aspiration cycles annually. When asked about the percentage of fresh embryo transfers in their center, 56.2% of respondents reported that these amounted to less than 50%.

Most respondents (98.9%) used US for monitoring OS cycles during ART treatment. HM was widely accepted and used by 420 (79.5%) of participants during any of the cycle monitoring visits.

**Figure 1:**
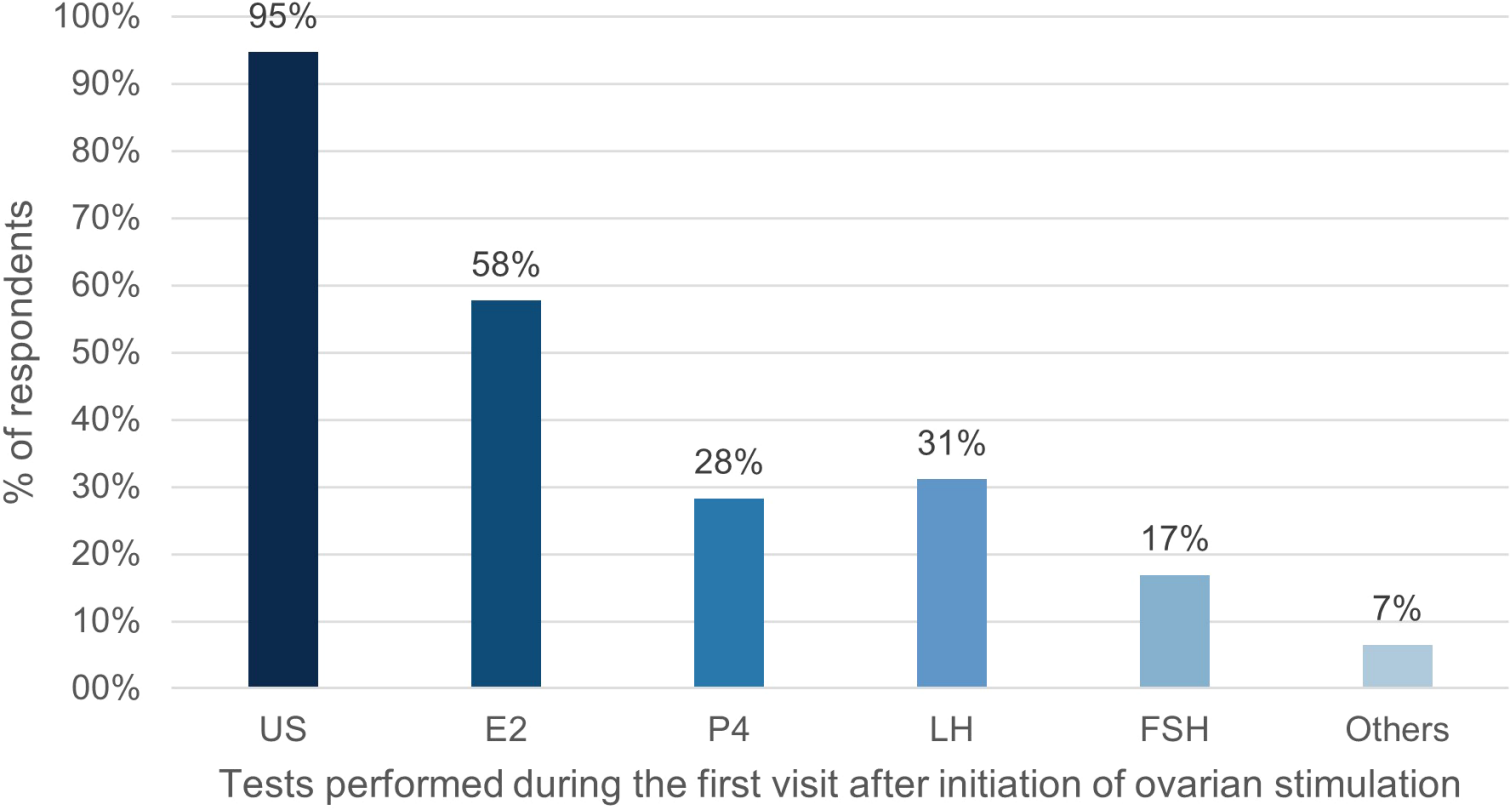
Percentage of respondents (%) that used tests during the first visit after initiation of ovarian stimulation. US, ultrasound; E2, oestradiol; P4, progesterone; LH, luteinizing hormone; FSH, follicle stimulating hormone.

**Figure 2:**
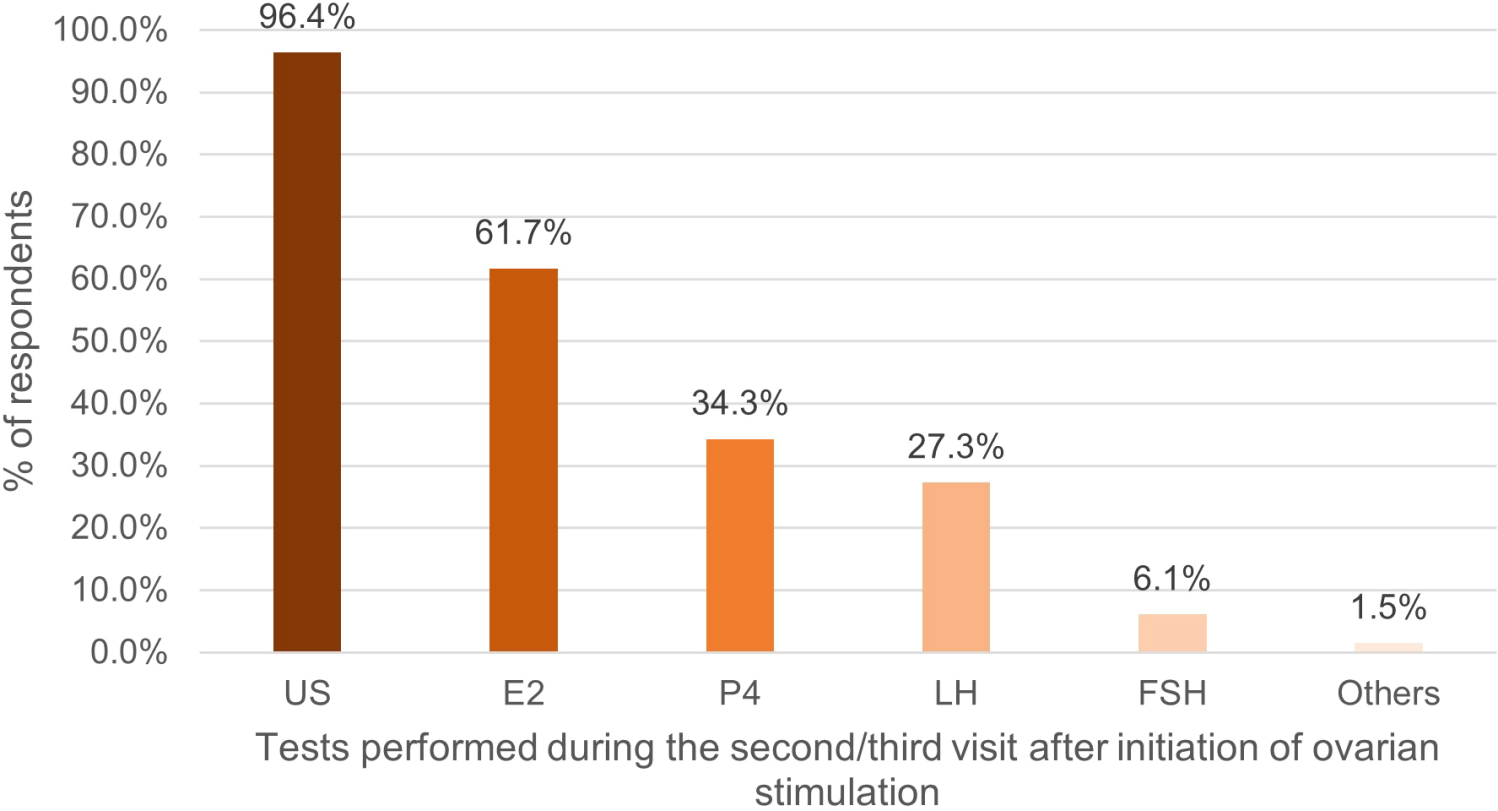
Percentage of respondents (%) that used tests during the second/third visit after initiation of ovarian stimulation. US, ultrasound; E2, oestradiol; P4, progesterone; LH, luteinizing hormone; FSH, follicle stimulating hormone.

E2 was the most frequently monitored hormone during the first and second/third clinic visit after the first gonadotropin injection. Notably, the proportion of respondents measuring E2 and P4 measurement increased in the second/third visit, while the proportion of those measuring follicle stimulating hormone (FSH) and LH decreased (**Figures 1**, **2**).

**Figure 3:**
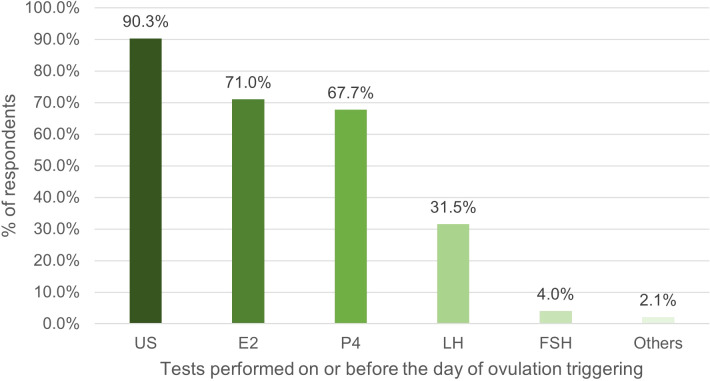
Percentage of respondents (%) that used tests on or before the day of ovulation triggering. US, ultrasound; E2, oestradiol; P4, progesterone; LH, luteinizing hormone; FSH, follicle stimulating hormone.

HM was most commonly performed on the day of, or day prior to final oocyte maturation, with 71% of respondents measuring E2. The number of respondents who measured P4 (67.7%) was twice that during the second/third visit. There was also an increase in the proportion of respondents measuring LH, from 27.3% in the second/third visit, to 31.5% in the visit on the day of, or day prior to ovulation triggering (**Figure 3**).

**Figure 4:**
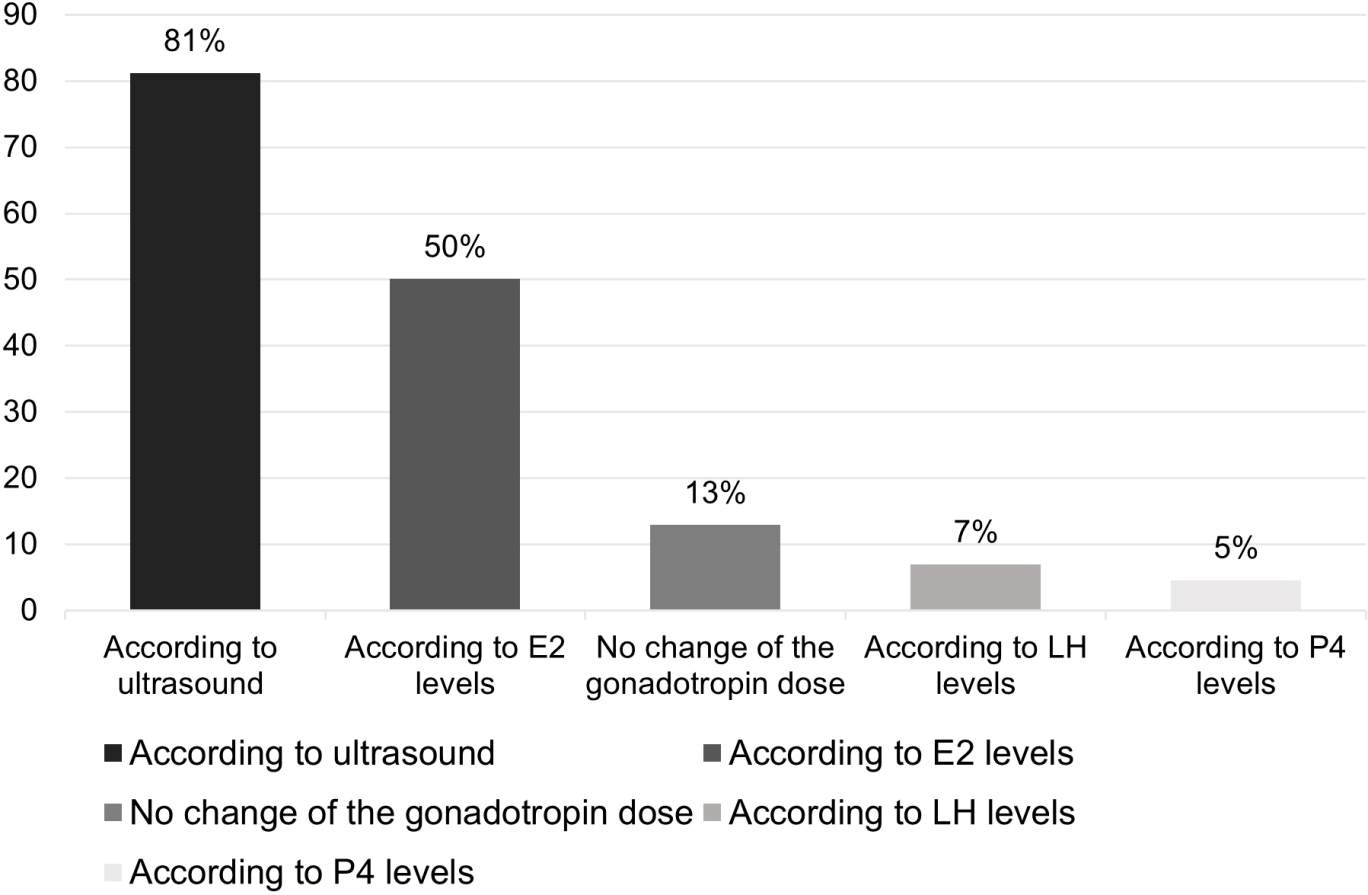
Percentage of respondents (%) that adjusted the dose of gonadotropin according to test results. E2, oestradiol; LH, luteinizing hormone; P4, progesterone.

A total of 87% respondents claimed that they adjusted the dose of gonadotropin during OS, with 81% of them adjusting the dose based on US findings and 61.7% adjusting it based on HM. In all, 50% of respondents adjust the dose based on E2 levels (**Figure 4**).

E2 monitoring was used by 74% of respondents for the prediction of ovarian hyperstimulation syndrome (OHSS). Among the respondents, 45% measured HM for timing the OT. While 55.4% of respondents did not check P4 during the luteal phase, 23.5% of them measured it in all patients or nearly all patients, and 21.1% measured it in some patients. Most respondents (60.7%) believed that hormones play an important role in monitoring ovarian response during OS, and 56% considered that HM is important to guide decision-making for the prevention of OHSS.

In the context of frozen embryo transfers, our study revealed diverse practices among respondents. A significant portion, 36.8%, reported utilizing HM for planning transfers in natural cycles. Additionally, 20.1% relied on LH urine test strips, 53.3% performed ultrasounds to confirm ovulation, and 42.3% conducted hormonal assessments and ultrasounds in cases of ovulation trigger in the modified natural cycle.

Concerning frozen embryo transfers in artificial cycles, 49.1% of respondents did not measure estrogen levels during the endometrial preparation, and 70% did not measure LH levels. However, 38.5% of respondents monitored progesterone levels for each patient during endometrial preparation. Notably, 97.3% of respondents conducted ultrasounds during artificial cycle preparation, and 37% assessed progesterone levels just before embryo transfer for nearly all patients.

## Discussion

A Cochrane review in 2014, and subsequently in 2021, concluded that there was no evidence to suggest that combined US and serum E2 monitoring is more efficacious than US monitoring alone in terms of clinical pregnancy rates and incidence of OHSS (8, 11). The chance of clinical pregnancy using US with E2 monitoring was 36% vs 31%-46% with US monitoring alone. There was no difference between the groups in the mean number of oocytes retrieved per woman. However, none of the six studies included in the review reported the primary outcome of live birth rate. Moreover, the evidence was of low quality for all comparisons. The randomization methods, allocation concealment, and blinding of the included studies were all unclear. Further, there were differences in treatment protocols and a lack of methodological descriptions in several studies. These limitations led to inaccuracy and potential bias. Most importantly, the conclusion was based on a meta-analysis of six studies, four of which were GnRH-agonist cycles and 2 were mixed GnRH-agonist and antagonist cycles. Therefore, it is not known whether the conclusion is valid only for GnRH-agonist cycles; moreover, there is no evidence for only GnRH antagonist cycles. A randomized prospective study in 2012 compared women undergoing IVF monitored only using US in comparison to those monitored with US combined with HM. No differences were found between the groups with respect to the duration of stimulation, number of ampoules of FSH used, E2 level on the day of OT, as well as embryo quality. The clinical pregnancy rates were not statistically different between the groups, 57.5% vs 40.0%, respectively (p=0.25), and there were no cases of OHSS reported in either group, which might relate to the small sample sizes of the study (n=63) (12).

Given that ovarian reserve markers do not always predict response to stimulation, an individualized treatment approach requires not only the proper selection of starting dose (13), but also a dose adjustment by combined monitoring using E2 and US. Low E2 levels after four to six days of gonadotropin stimulation were reported to lead to a high likelihood of cycle cancellation and lower pregnancy outcomes in previous studies (14, 15). However, the ESHRE 2019 guideline does not support changing gonadotropin dose during OS in the mid-stimulation phase, citing lack of evidence (6), despite this guideline our survey demonstrated that 87% of respondents claimed they routinely adjust the dose of gonadotropin during treatment.

US monitoring measures follicular development, whilst a patient’s serum E2 concentration is a marker of follicular function (16). Consequently, adequate E2 levels indicate follicular maturity, while very high levels indicate an increased risk of OHSS (6, 17). This risk might be the indication for the significant number of respondents measuring serum E2 being greatest on the day of or just prior to OT, even though it is well known that E2 might not be the only parameter to be considered when predicting OHSS (18).

Approximately one-third of respondents also regularly measured LH. Physiological levels of LH are important for follicular development and abnormal levels lead to abnormal follicular development (19, 20). LH induces a dose-dependent production of E2, and this is critical for endometrial preparation for embryo implantation. A minimal level of LH, described as the ‘LH threshold,’ is necessary for successful pregnancy. For instance, a low serum LH concentration on the day of ovulation triggering is associated with reduced reproductive outcomes in GnRH agonist fresh embryo transfer cycles (21). However, higher levels have an adverse impact on the endometrium (19, 22). LH above a certain threshold may lead to atresia of less mature follicles (22). The serum LH concentration on the day of ovulation triggering is also an indicator to change from prescribing a GnRH-agonist to an hCG trigger for induction of final oocyte maturation, for better pregnancy outcome in fresh embryo transfer cycles (21). Similarly, premature P4 elevation can have a negative impact on the outcome of ART (22). This might be one of the reasons there was an increase in the proportion of respondents measuring P4 from 34.3% in the second/third visit to 67.7% on or before the day of OT. Late-follicular phase P4 elevation (PE) occurs in up to 46.7% of fresh IVF cycles (23). The dose of gonadotropins is one of the factors associated with PE (23). Elevated P4 levels affect the endometrium and the window of implantation (22). This might lead to embryo-endometrium asynchrony and decrease the fresh embryo implantation rates (24). Elevated progesterone level during the late follicular phase is an independent risk factor affecting the clinical pregnancy rate and live birth rate after fresh embryo transfers (25). High concentrations of P4 at the beginning of an IVF antagonist cycle and on the day of OT have also been reported to be associated with a lower probability of clinical pregnancy (26, 27). Decreasing the stimulation dose during the late follicular phase of OS could reduce PE (22). Prolongation of OS beyond the optimal criteria for final oocyte maturation should be avoided (22). P4 monitoring is thus important for making these decisions. A study showed that insufficient P4 control (>1.5 ng/mL) on the day of OT is related to poor delivery rates (28). A large retrospective study proposed that a P4 concentration of 1.5 ng/mL as being the threshold for poor responders, 1.75 ng/mL for intermediate responders, and 2.25 ng/mL as being the threshold for high responders’ chance of pregnancy (29).

Most respondents in the survey (60.7%) believe that HM is important for monitoring the ovarian response during OS, and 56% consider that it is important to guide decision-making for preventing OHSS. However, the Cochrane 2021 meta-analysis reported that there was no evidence of a difference in the incidence of OHSS between the groups with US or US with HM monitoring, 8% in comparison to 4% respectively (8). The real-world data indicate that the incidence of cases of OHSS requiring hospitalization is 2% (30). As close to 2 million ART cycles are reported each year worldwide the total number of patients hospitalized for OHSS is likely to be an enormous burden on the health system. With nearly 2 million ART cycles documented annually across the globe, the potential surge in hospitalizations due to OHSS represents a significant strain on healthcare systems. Hence, it is proposed that it is good clinical practice to use HM to minimize the risk of OHSS. This recommendation holds, notwithstanding the effectiveness of the GnRH agonist and freeze-all strategy in reducing OHSS risk. Hence it is proposed that it is good clinical practice to use HM to minimize the risk of OHSS (8).

While in ovarian stimulation protocols the guidelines and reviews do not recommend routine HM, in the context of a natural cycle frozen embryo transfer, evidence demonstrate that correctly identifying ovulation is challenging and that hormonal measurements of LH, E2, and P4 are essential for properly planning the embryo transfer timing (31).

Concerning hormonal monitoring during artificial cycle frozen embryo transfer, HM is not supported by evidence during endometrial preparation (32–34). However, evidence show that a minimal serum progesterone level is necessary to ensure an optimal endocrine environment for embryo implantation and early pregnancy (35). Measuring progesterone levels, the day before embryo transfer also allows for individualizing luteal phase support, and several studies have demonstrated the effectiveness of progesterone rescue in women with low progesterone levels around the day of embryo transfer (36, 37).

The data of this survey reflect the global practice of HM during IVF cycles. Despite the guidelines recommending no HM and no adjustment of gonadotropin dose, it is clear that the majority of ART specialists consider HM as essential, and ~80% across the globe use HM along with US for monitoring OS, especially for the prevention of OHSS, regardless of the additional costs it can imply. Despite the fact of not being backed up by guidelines and other type of recommendations, there is extensive evidence supporting individual hormonal measurements such as basal LH and late follicular progesterone, during the ovarian stimulation cycles. Good quality studies are necessary to document the value of HM during OS with respect to dose adjustment, OT, and prevention of OHSS.

## Data availability statement

The datasets presented in this study can be found in online repositories. The names of the repository/repositories and accession number(s) can be found below: https://ivf-worldwide.com/survey/blood-hormone-monitoring-in-controlled-ovarian-stimulation/results-blood-hormone-monitoring-in-controlled-ovarian-stimulation.html.

## Ethics statement

Ethical approval was not required for the study involving humans in accordance with the local legislation and institutional requirements. Written informed consent to participate in this study was not required from the participants or the participants’ legal guardians/next of kin in accordance with the national legislation and the institutional requirements.

## Author contributions

NS-G: Writing – original draft, Writing – review & editing. RH: Conceptualization, Formal analysis, Methodology, Writing – review & editing. AR: Conceptualization, Formal analysis, Methodology, Writing – review & editing. VV: Conceptualization, Formal analysis, Methodology, Writing – review & editing. NP: Conceptualization, Formal analysis, Methodology, Supervision, Writing – review & editing.
